# Discrimination and Violence Due to Diversity of Sexual Orientation and Gender Identity: Explanatory Variables

**DOI:** 10.3390/ijerph18073638

**Published:** 2021-03-31

**Authors:** Fernández-Antelo Inmaculada, Cuadrado-Gordillo Isabel

**Affiliations:** Department of Psychology and Anthropology, University of Extremadura, 06071 Badajoz, Spain; cuadrado@unex.es

**Keywords:** dating violence, homosexuality, aggressive attitudes, moral disengagement

## Abstract

Knowledge of the processes of aggression and victimization in couple relationships cannot be approached through the treatment of single variables. It needs a multidimensional perspective that establishes a web of relationships between variables of different types. The objectives of the present study were: (i) to explore the interrelationships between and interdependence of empathy, moral disengagement, homophobic attitudes, and prejudice as explanatory variables of discrimination and violence towards couples due to gender issues; and (ii) to delimit predictive indicators of the manifestation of aggressive attitudes and prejudices towards homosexual couples. The sample comprised 778 young people of ages 18 to 24 years (M = 19.9; SD = 1.6). Through the use of four instruments, it was found that empathy is a strong protector against homophobic attitudes, while moral disengagement is a predictor of aggressive attitudes towards same-sex couples. The results make it possible to delimit homophobic profiles and obtain predictive indicators that will be key elements in the design of programs and measures to prevent violence towards couples for reasons of gender.

## 1. Introduction

The government report about the evolution of hate crimes in Spain [1] indicates that the three areas with the greatest numbers of recorded incidents are those of “ideology”, “racism/xenophobia”, and “sexual orientation/gender identity”, which represent 37.3%, 33.2%, and 16.2% of the total of hate crimes, respectively. With regard to the last datum, which includes discriminatory attitudes towards the LGTBI community, homophobia is considered a hostile attitude which conceives of and points to homosexual orientation as being contrary, inferior, or abnormal, and the people who practise it as being sinners, sick, bad, delinquents, criminals, or unbalanced, even stripping them of their condition as human beings [2].

In recent years, there have been studies which have focused on the possible affective variables that may explain the deployment of intimidatory attitudes towards LGTBI couples. One of the variables most explored is empathy, understood as a multidimensional construct that includes both affective and cognitive components [3]. Empathy has been shown to be negatively related to aggression [4], to have a modulating function in both prosocial and aggressive behaviour [5], to be a strong predictor of openness to diversity [6], and to reduce prejudices and foster prosocial behaviour [7]. Other studies corroborate this negative relationship between prejudice and empathy [8], with homophobic attitudes registered in persons with low levels of empathy [9], both in the heterosexual adolescent population [10] and in homosexual or bisexual people [11].

Nevertheless, despite the empirical evidence for this inverse relationship between empathy and homophobia, other studies nuance this connection, indicating that the manifestation of intimidatory or violent attitudes is more related to low levels of the affective components of empathy without having an impact on the cognitive components [12]. Other researchers note the weakness of the relationship between empathy and aggression, with only a minimal part of the variation in aggression being explicable by empathy [13]. These discrepancies highlight the importance of the distinction between cognitive and affective empathy linked to the intimidation of others [14,15].

Another of the variables included in this study of violence against homosexual couples is prejudice, understood in this context as being a discriminatory attitude directed towards a person based on their homosexual identity [16]. In this regard, a positive correlation has been shown between levels of prejudice and social distance towards gays and lesbians, i.e., the greater the prejudice, the greater the level of homophobia and the social distancing wanted with these two groups.

The manifestations of these forms of prejudice include real or symbolic violence, analogous to other forms of exclusion [17]. In this sense, a parallel between racial prejudice and homophobia has also been confirmed [18]. The attitude of open condemnation involved in both types of prejudice has tended to disappear to be replaced by subtle or disguised rejection. In the case of homophobia, this is characterized by softening the signs of discrimination aimed towards gays and lesbians, and by non-acceptance of the normalization of the life and equality of LGTBI people. This is thus confirmation of the existence of forms of both overt and subtle homophobia that follow the same lines as in racial prejudice [18]. Three profiles have been identified [19]: *egalitarian*, scoring low in overt and subtle prejudice, and characterized by low rejection of intimacy and by expression of positive emotions towards the out-group; *subtle*, scoring low in overt prejudice but high in subtle prejudice, and revealing little rejection of intimacy or denial of positive emotions towards the out-group; and *fanatic*, with high scores on both scales that are characterized by strong rejection of intimacy and denial of positive emotions towards the out-group.

Some studies have addressed the identification of these profiles in analyses of violence towards LGTBI couples. With undergraduates forming their sample, Quiles et al. [18] detected 43% with an egalitarian profile, 20% with a subtle profile, and 25% with a fanatic profile. With a sample consisting of teachers however, Testor et al. [20] found substantially different proportions, with the respective percentages being 87.5%, 9.2%, and 3.3%.

With respect to the influence of the gender variable on prejudicial attitudes towards homosexual couples, some researchers found that men are more homophobic than women, and that greater negativity is repeatedly observed towards gays than towards lesbians [21]. Homophobia becomes a reason for exclusion and aggression when men do not behave in accordance with the hegemonic masculinity profile typical of traditional masculinities [22]. However, other researchers propose a construct such as ‘cultural homophobia’ whose pillars are based on the universality of heterosexuality [23]. In this sense, it is understood that homophobia is formed by social guidelines that highlight the moral supremacy of heterosexuality over homosexuality. The bases of patriarchy and heteronormativity that are still fundamentally in force in Western societies explain the maintenance and to some extent the rise of sexist and homophobic attitudes towards gays and lesbians.

Another variable that has been associated with hostile and aggressive attitudes is moral disengagement, but there have been few studies of its influence on the appearance and permanence of abusive attitudes and behaviours towards LGTBI couples. According to some researchers [24], high levels of moral disengagement can be predictive of general attitudes of racist and homophobic harassment. Other studies also point to mechanisms of moral disengagement being used to justify hurt caused to the LGTBI community [25].

Some moral disengagement mechanisms are used more than others in manifestations of antisocial behaviour or harassment. Stiths & Narváez [26] note the priority use of moral justification, while Canchila, Hoyos & Valega [27] note rather the preferential use of the displacement and diffusion of responsibility, the distortion of consequences, and attribution of blame (victim blaming). Other studies indicate the frequent use of moral justification, euphemistic language, advantageous comparison, and displacement and diffusion of responsibility [28,29].

Explanations of the manifestation of violent attitudes towards homosexual couples of either sex that consider the influence of just a single variable can provide no more than limited knowledge of the processes of aggression and victimization involved in this type of violence. To define effective guidelines for prevention and intervention regarding discrimination against people on the basis of gender requires knowledge of how different variables may be interdependent and interrelated. Indeed, linking such variables as moral disengagement and empathy has yielded some preliminary results that point to the existence of a negative relationship between them [26,30,31,32]. Likewise, it has been confirmed that, while at low levels of moral disengagement, empathy and aggression are negatively related, at high levels this relationship is not significant [15].

The disparity of the results and the one-dimensional treatment with which the influence of certain variables on the processes of aggression towards LGTBI couples has been analysed suggest the need to address this topic multidimensionally, so as to establish whether there is a web of relationships between moral, emotional, cognitive, and psychological variables. In this sense, the present study’s objectives were the following: (i) to explore the interrelationships between and interdependence of empathy, moral disengagement, homophobic attitudes, and prejudice as explanatory variables of discrimination towards couples due to gender issues; and (ii) to delimit predictive indicators of the manifestation of aggressive attitudes towards homosexual couples.

## 2. Materials and Methods

### 2.1. Participants

Incidental non-probabilistic convenience sampling was used to form a set of participants formed by 778 young (ages 18 to 24 years, M = 19.9, SD = 1.6) people (61.4% women). They were recruited from students at various secondary education schools of the Region of Extremadura (Spain) who were in Lower Secondary (*n* = 145), Upper Secondary (*n* = 69), and Baccalaureate (*n* = 43) cycles, and from undergraduates at the University of Extremadura (*n* = 521) studying Pre-Primary Education, Primary Education, and Psychology & Social Education. The sample is composed of undergraduates come from very different geographical areas and belong to very diverse social strata. Specifically, young people who attend Lower Secondary generally come from rural areas and peripheral neighborhoods of the city that are characterized by a medium-low socioeconomic level. The sample of participants belonging to Upper Secondary and Baccalaureate is drawn from educational centers located in central urban areas with a medium-high socioeconomic level. Finally, university students come from both rural and urban cities and from families with very different purchasing power and cultural levels. In this way, the entire diversity of the population that defines the region is covered. Regarding the ethnic variable, the entire sample are adolescents born in Extremadura. This region is not characterized by a great diversity of races or ethnicities and when there are, they generally do not pursue higher education. Another variable to consider is sexual orientation. The sample is made up of 19 young people who declare themselves homosexual, 699 heterosexual and 60 bisexual.

It should be clarified that the initial sample consisted of 855 subjects. However, those cases in which adolescents did not answer all the questions have been excluded, leaving a total of 778 young people.

### 2.2. Instruments

Davis Interpersonal Reactivity Index (IRI). The Davis Interpersonal Reactivity Index (IRI) is a questionnaire with 28 items organized into four 7-item subscales (Perspective Taking, Fantasy, Empathic Preoccupation, and Discomfort), each one allowing different dimensions of (cognitive and affective) empathy to be assessed. The outstanding characteristic of this instrument is that it allows measurement of both the cognitive aspect and the emotional reaction of the individual when adopting an empathic attitude [33,34]. Specifically, the Perspective Taking and Fantasy subscales are aimed at assessing cognitive empathy and the Empathic Preoccupation and Discomfort subscales are aimed at the emotional dimension of empathy. The responses are given on a 5-point Likert scale, with 1 being strongly disagree and 5 strongly agree. With respect to the reliability of the instrument, the values of Cronbach’s α obtained in the four subscales were from 0.70 to 0.77, values even higher than those reported by Mestre, Samper & Frías [5] for their Spanish adaptation of the questionnaire. The reliability obtained in the cognitive dimension was α = 0.72, and in the affective dimension α = 0.74.

Mechanisms of Moral Disengagement Scale (MMDS). To measure moral disengagement, the Mechanisms of Moral Disengagement Scale (MMDS) was used [35]. This instrument consists of 32 items with responses given on a 5-point Likert scale, with 1 being strongly disagree and 5 strongly agree. It is organized into eight 4-item factors which correspond to the eight mechanisms of moral disengagement: moral justification, euphemistic language, advantageous comparison, displacement of responsibility, diffusion of responsibility, distortion of consequences, attribution of blame, and dehumanization. In turn, these eight mechanisms are grouped into four dimensions or loci: a behavioural locus which includes three disengagement mechanisms—moral justification, euphemistic language, and advantageous comparison; an outcome (effects) locus which includes one disengagement mechanism—distortion of consequences; an agency locus which includes two disengagement mechanisms—displacement and diffusion of responsibility; and a recipient (victim) locus which includes two disengagement mechanisms—dehumanization and attribution of blame. With respect to the general reliability of the instrument, its overall Cronbach’s α was 0.81, and the α values for the four loci separately ranged from 0.78 to 0.84.

Modern Homophobia Scale (MHS). To measure homophobia, the Modern Homophobia Scale (MHS) of Raja & Stokes [36] was used. This consists of two subscales—the 22-item MHS-G which measures attitudes towards the gay community and which has a reliability index of α = 0.91, and the 24-item MSH-L which measures attitudes towards the lesbian community and has a reliability index of α = 0.89. The responses are on a 5-point Likert scale, with 1 representing the greatest disagreement and 5 the greatest agreement. Each subscale comprises three factors (personal distress, deviance/changeability, and institutional homophobia) corresponding to measures of personal and institutional homophobic attitudes towards gays and lesbians. However, in this study, the analysis by factors has not been considered, but rather the global value obtained in each of the subscales. Likewise, this instrument allows obtaining a global value of homophobia, whose level of reliability in this study reaches a value of α = 0.85.

Subtle and Overt Homophobia Scale. The Subtle and Overt Homophobia Scale of Quiles et al. [18] is a 17-item questionnaire assessing attitudes towards homosexuality. Each item is scored on a 7-point Likert scale with 1 representing the greatest disagreement and 5 the greatest agreement. It has two subscales: overt and subtle homophobia. Their respective Cronbach’s α reliability indices are α = 0.73 and α = 0.70. Application of the questionnaire allows three profiles to be distinguished: egalitarian, with low scores on both overt and subtle homophobia; subtle, with low scores on overt homophobia but high on subtle homophobia; and fanatic, with high scores on both subscales.

### 2.3. Procedure

Prior to handing out the questionnaires to the participants, permission was requested from the Regional Educational Administration for access to the secondary schools so as to pass the questionnaires to pupils in them of at least 18 years in age who wished to participate in the study. In the undergraduate context, authorization was requested from the deans of the universities and from the teaching staff to give up time from their classes so that the students could complete the questionnaires. Once all the permissions had been obtained, one of the researchers took on the responsibility of visiting all the classrooms, handing out the questionnaires, and resolving any doubts that arose. At all times, the participants were informed of the research objectives and of the guarantee of confidentiality of the data.

### 2.4. Analysis

After a preliminary analysis of the descriptive data, a correlation analysis was applied to determine the relevance and interconnection of the variables involved. Once the importance of the prejudice variable had been detected, the discriminatory profiles defining the participants were identified and their prevalence analysed. An analysis of variance (ANOVA) was then applied to investigate whether having one or another of the profiles implies the use of different moral disengagement mechanisms, the manifestation of variable homophobic attitudes towards homosexual couples, or different levels of empathy or the prioritization of its cognitive and affective dimensions. Finally, a hierarchical multiple regression analysis was used to reveal any variables predictive of the appearance of discriminatory attitudes towards gay and lesbian couples.

## 3. Results

The preliminary correlation analysis showed the variables under study to be strongly related. Homophobia (calculated from the global MHS value), prejudice (calculated from the global Subtle and Overt Homophobia Scale value), and moral disengagement were positively correlated, and all of these variables were negatively correlated with empathy (Table 1).

The results revealed a strong interrelationship between the manifestation of homophobic attitudes towards homosexual couples and the existence of prejudices (Table 1). Three types of profile were identified according to the type of prejudice towards the gay and lesbian collective the participants present: egalitarian (*n* = 541), subtle (*n* = 70), and fanatic (*n* = 146). These different profiles condition the use of moral disengagement mechanisms and the participants’ levels of both cognitive and affective empathy (Table 2). But while they condition homophobic attitudes towards gay couples, they have no significant influence on those towards lesbian couples (Table 2).

The demonstration that moral, psychological, and personal variables are related to the manifestations of homophobic attitudes towards gay and lesbian couples justified the analysis of those variables’ predictive value by means of a hierarchical regression analysis. This was done by constructing a hierarchy of 5 models that include the independent variable gender together with the different dimensions of prejudice, moral disengagement, and empathy. Model 1 includes gender and prejudice. Model 2 adds moral disengagement mechanisms, both overall and in its four loci (behavioural locus, outcome locus, agency locus, recipient locus). Model 3 further adds cognitive and affective empathy. Model 4 adds to this the interaction between moral disengagement and cognitive empathy. Finally, Model 5 instead adds the interaction between moral disengagement and affective empathy to the variables of Model 3.

The results of the regression analysis indicate that the variable gender is a predictor of homophobic attitudes (Table 3), and that the profiles of fanatic (to a greater extent) and subtle (to a lesser extent) prejudice are also. Specifically, it is found that boys show a greater predisposition to manifest maladaptive attitudes and prejudices towards homosexual people. Likewise, moral disengagement is a variable that could explain the existence of homophobic attitudes, especially when there are high scores in mechanisms associated with the behavioural, outcome, and agency loci (Table 3). Model 3 indicates that low levels of empathy, both cognitive and affective, are indicative of a greater likelihood of discriminatory and abusive attitudes towards gay and lesbian couples.

The interaction between Models 2 and 3 shows the linkage between moral disengagement and the affective and cognitive dimensions of empathy (Models 4 and 5, Table 3). One finds that respondents who present high levels of moral disengagement and low levels of empathy (cognitive and/or affective) constitute a group who could to a large extent manifest attitudes of incomprehension, discrimination, and violence towards homosexual couple relationships. At the same time, the gender variable interacts with the dimensions of empathy and prejudice profiles, so that boys with subtle and fanatical profiles present low levels of empathy and this puts them at risk of showing homophobic attitudes.

In synthesis, the results provide indicators that delineate the following predictive profile—persons with high levels of subtle and overt homophobic prejudices, who are lacking in both cognitive and affective empathy, and who resort to mechanisms of moral disengagement in order to justify and validate their aggressive attitudes towards gay and lesbian couples (Table 3).

## 4. Discussion

The interrelationship between the manifestation of homophobic attitudes towards homosexual couples and the existence of prejudices is consistent with findings of previous studies that individuals with a high level of prejudice tend to manifest more attitudes that are homophobic [21,37]. In this regard, various theoretical positions have been taken to provide a possible explanation for the predictive value that prejudice towards minority groups has. On the one hand, Tajfel’s social identity theory [38] establishes that the mere categorization into different groups will accentuate their differences. And on the other, social dominance theory [39] postulates that every society is organized around the principle of hierarchical ordering, and that prejudice, homophobic prejudice in the present case, would justify social inequality, and heterosexual dominant groups would enjoy the privileges offered by their position.

In coherence with previous research [18], the results for the prejudice variable showed significant differences between subtle and overt homophobia, confirming the existence of three prejudice profiles: egalitarian, subtle, and fanatic. The present study’s description of these profiles not only takes account of the scores measuring discrimination expressed towards gay and lesbian couples, but also each of the respondents’ use of moral disengagement mechanisms and levels of cognitive and affective empathy. In particular, the fanatic group is characterized by the use of moral disengagement mechanisms mainly associated with the recipient locus (dehumanization and the attribution of blame), but also to a lesser extent with the outcome locus (displacement and diffusion of responsibility). They also have very low levels of empathy, with the cognitive being slightly, although not significantly, higher than the affective. These results are coherent with those of Blair [12] relating empathy with the manifestation of intimidatory or violent behaviour. As Ortega, Sánchez & Menesini [40] indicate, the lack of affective empathy leads to moral disengagement, generating in the present case homophobic attitudes.

The subtle group is characterized by their non-use of moral disengagement mechanisms, perhaps due to the influence of cognitive and affective empathy. A notable result is that this group’s scores on homophobia towards lesbians are similar to those of the egalitarian and fanatic groups. In this regard, some research has found that heterosexual women tend to express more positive attitudes towards gays than towards lesbians [36,41], while heterosexual men express more positive attitudes towards lesbians than towards gays [42]. As Barra [43] points out, the latter case, the rejection of gays, could be a way for heterosexual men to protect their social status and the power associated with the male condition. These gender differences are also confirmed in our study, where boys tend to show homophobic attitudes and prejudices to a greater extent than girls, especially when the couple is made up of men.

Finally, the egalitarian group scores high in both cognitive and affective empathy and stands out for egalitarian attitudes towards the gay community, although towards lesbians its scores are similar to those of the other two groups. Evidently, they do not use moral disengagement mechanisms due to their lack of attitudes of rejection and their high empathy towards gay and lesbian couples.

Our results regarding the prevalence in each of the groups are in line with those of Quiles et al. [18]. However, while the proportionality is the same in the two studies, the levels of homophobia that we observed are significantly lower. This difference in homophobic attitudes may be attributable to the effectiveness of the legislation in Spain from 2003 to date promoting equality without discrimination based on sexual orientation (Ley 62/2003), and to the legal recognition of same-sex marriage (Ley 13/2005), among other regulations. Another possible explanation lies in the present sample’s academic profile, since most are undergraduates doing degree courses related to Education and Psychology, subjects that are sensitive to non-discrimination.

The relationship between moral disengagement and homophobic prejudice acquires a predictive value in showing that those presenting more discriminatory and aggressive attitudes towards gay and lesbian couples more frequently resort to using justifications and moral arguments that offer a kinder and less obvious view of their prejudices. This finding is consistent with previous research that observed this positive relationship between moral disengagement and intimidatory and antisocial behaviour in general [40], and more specifically with studies focused on the manifestation of homophobic attitudes [24]. Nevertheless, there has been little research into the influence of moral variables on the adoption of aggressive and discriminatory attitudes towards LGTBI couples, so that it is hard to find previous work with which to compare this aspect of the present results. In this sense, Sahlman [25] reports some preliminary results that are in line with those obtained here on the part that can be played by moral disengagement in justifying abusive behaviour towards the gay and lesbian collective.

With respect to the empathy variable, our results reveal it to be inversely related to homophobia, behaving as an inhibitor of antisocial attitudes towards gay and lesbian couples. Previous studies too have found a negative relation of empathy with aggression and intimidatory attitudes [4,7], with prejudice [8], and with homophobic attitudes specifically [9,10,11], so that it constitutes a strong negative predictor of hostile attitudes towards the gay and lesbian collective.

With respect to the analysis of the conjoint effect on homophobia of moral disengagement and empathy, our results indicate that both cognitive and affective empathy correlate negatively with moral disengagement, and that the latter correlates positively with homophobia. This finding is coherent with previous studies that analysed the conjoint effect of these variables in the contest of violence and abuse among peers [40]. High empathy constitutes therefore a protective factor, and high moral disengagement a risk factor, for homophobic attitudes towards gay and lesbian couples.

## 5. Conclusions

The contributions of this study to knowledge of the explanatory variables for the manifestation of aggressive attitudes towards gay and lesbian couples lie in its adoption of a multidimensional approach which included moral and affective variables as well as their interaction. The delimitation of homophobic profiles and the determination of predictive indicators will undoubtedly be key elements in the design of programs and measures for the prevention of violence against couples based on gender. Throughout this work, we have been able to verify how cognitive and affective empathy, in their relationships with attitudes of rejection and discrimination towards gay and lesbian couples, both constitute strong protectors against homophobic attitudes. The gender variable has been shown to be another predictive indicator of homophobic attitudes especially directed towards gay couples.

The study has confirmed the direct link between moral disengagement and homophobia, in the same way as such a link had been demonstrated in studies on harassment, intimidation, and antisocial attitudes, i.e., moral disengagement predicts levels of homophobia. Likewise, the relationship found between moral disengagement and empathy concerning homophobia has confirmed that lack of empathy leads to the use of moral disengagement mechanisms, and that these in turn generate homophobic attitudes. 

Possible political implications follow from the results of this study. On the one hand, the need to strengthen gender policies is evident. If boys are more homophobic than girls, it is found that the current social structures are still based on a heterosexual patriarchal system. On the other hand, it is essential to bet on policies to protect diversity. In the 21st century, it is still free to discriminate, offend, exclude or ridicule people for the sole fact of being different from others, in this case, for showing sexual orientations other than heteronormativity.

## 6. Limitations

The definition of profiles of young people with a tendency towards discrimination and violence towards gays and lesbians constitutes an element of great value for the delimitation of prevention programs towards this type of violence. However, defining these profiles through a cross-sectional study may present some limitations. It would be advisable to initiate longitudinal studies to confirm or adjust the profiles presented in this study. Another possible limitation is in the composition of the sample. It could be assumed that university students who study social careers related to the training of teachers and psychologists could present greater sensitivity towards the manifestation of attitudes of respect and tolerance towards diversity, than students of professional training or high school whose vocational orientation or not is defined or could be guided towards highly normative trades.

## Figures and Tables

**Table 1:** Correlations between the study variables.

Variables	1	2	3	4	5	6	7	8	9
1. Homophobia	-	0.41 ***	0.46 ***	0.37 ***	0.29 ***	0.32 ***	0.52 ***	−0.32 ***	−0.39 ***
2. Prejudice		-	0.44 ***	0.38 ***	0.32 ***	0.34 ***	0.41 ***	−0.29 ***	−0.33 ***
3. Behavior locus			-	0.31 ***	0.36 ***	0.28 ***	0.46 ***	−0.36 ***	−0.31 ***
4. Outcome locus				-	0.31 ***	0.30 ***	0.45 ***	−0.34 ***	−0.28 ***
5. Agency locus					-	0.26 ***	0.42 ***	−0.32 ***	−0.34 ***
6. Locus of the recipient						-	0.44 ***	−0.26 ***	−0.35 ***
7. Moral disengagement							-	−0.34 ***	−0.38 ***
8. Cognitive empathy								-	0.029 ***
9. Emotional empathy									-

*** *p* < 0.001

**Table 2:** Variability in homophobic attitudes, moral disengagement mechanisms, and levels of empathy according to prejudice profile.

Variables	Equal	Subtle	Fanatic	F
Gay homophobia	2.32 (0.16)	2.69 (0.18)	3.09 (0.15)	8.23 *
Lesbian homophobia	2.93 (0.17)	2.87 (0.12)	3.12 (0.18)	3.87
Behavior locus	1.75 (0.23)	2.51 (0.19)	3.29 (0.24)	12.16 **
Outcome locus	1.36 (0.29)	2.12 (0.22)	3.31 (0.21)	11.86 **
Agency locus	2.18 (0.17)	2.24 (0.21)	3.06 (0.19)	9.05 *
Locus of the recipient	1.63 (0.20)	2.16 (0.24)	3.45 (0.27)	12.04 **
Moral disengagement	1.71 (0.19)	2.18 (0.20)	3.19 (0.22)	11.46 **
Cognitive empathy	3.78 (0.14)	3.21 (0.16)	2.33 (0.22)	9.48 *
Emotional empathy	3.48 (0.19)	3.09 (0.17)	2.19 (0.18)	9.71 *

Standard errors are in parentheses. Degrees of freedom for each analysis = 2. * *p* < 0.05. ** *p* < 0.01.

**Table 3:** Predictors of homophobic attitudes towards LGTBI couples.

Predictor	Δ*R*^2^	β	*T*
Step 1	0.11 **		
Gender		0.43	11.89 **
Prejudice		0.34	9.87 *
Step 2	0.19 **		
Gender		0.36	9.62 *
Prejudice		0.37	10.04 **
Behavior locus		0.49	12.28 **
Outcome locus		0.42	11.74 **
Agency locus		0.33	9.34 *
Locus of the recipient		0.20	7.01
Moral disengagement		0.38	10.49 **
Step 3	0.16 *		
Gender		0.32	9.27 *
Prejudice		0.34	9.71 *
Behavior locus		0.43	11.86 **
Outcome locus		0.38	10.53 **
Agency locus		0.29	8.67 **
Locus of the recipient		0.17	3.78
Moral disengagement		0.35	9.91 *
Cognitive empathy		−0.29	8.46 *
Emotional empathy		−0.32	9.19 *
Step 4	0.08 **		
Gender		0.28	8.31 *
Prejudice		0.32	9.24 *
Behavior locus		0.41	11.08 **
Outcome locus		0.35	9.78 *
Agency locus		0.26	7.84 *
Locus of the recipient		0.15	2.91
Moral disengagement		0.34	9.83 *
Cognitive empathy		−0.27	8.35 *
Emotional empathy		−0.29	8.53 *
Moral disengagement X Cognitive empathy		−0.26	8.30 *
Step 5	0.06 **		
Gender		0.24	7.14 *
Prejudice		0.35	9.64 *
Behavior locus		0.39	10.62 **
Outcome locus		0.32	9.58 *
Agency locus		0.25	8.17 *
Locus of the recipient		0.17	3.95
Moral disengagement		0.31	9.20 *
Cognitive empathy		−0.25	8.19 *
Emotional empathy		−0.28	8.47 *
Moral disengagement X Emotional empathy		−0.29	8.62 *
Total R^2^	0.22 **		

* *p* < 0.05; ** *p* < 0.01.
